# Integrative Approaches to Treating Cellular Senescence in Kidney Disease

**DOI:** 10.1002/advs.202519392

**Published:** 2026-03-14

**Authors:** Tomoka Misawa, Amruta A, LaTonya J. Hickson, Joy Wolfram

**Affiliations:** ^1^ School of Chemical Engineering The University of Queensland Brisbane Queensland Australia; ^2^ School of Chemistry, Chemical Engineering and Biotechnology Nanyang Technological University Singapore; ^3^ Division of Nephrology and Hypertension, Department of Medicine Mayo Clinic Jacksonville Florida USA; ^4^ Center For Regenerative Biotherapeutics, Mayo Clinic Jacksonville Florida USA; ^5^ Australian Institute for Bioengineering and Nanotechnology The University of Queensland Brisbane Queensland Australia

**Keywords:** cellular senescence, combination therapy, extracellular vesicle, kidney disease, longevity protein, mesenchymal stem cell

## Abstract

Cellular senescence in the kidney plays a crucial role in the progression of acute kidney injury and chronic kidney disease. Therapeutic approaches targeting senescent cells, such as small molecule senolytic and senomorphic drugs, display efficacy in preclinical models. However, such drugs pose a risk of adverse effects and only partially mitigate disease progression, highlighting the need for new therapeutic approaches that more comprehensively and safely address disease pathways in aging kidney disease. This review discusses the potential of extracellular vesicles and longevity proteins, such as α‐klotho and silencing information regulator 2‐related enzyme 1 (SIRT1), in regulating cellular senescence and alleviating kidney fibrosis. Particularly, combination therapy that simultaneously targets inflammation, tissue damage, and senescence is promising for kidney disease, given the potential to synergistically overcome the limitations of current unimodular treatment modalities and pave the way for more effective management of kidney disease. This review highlights the mechanisms of cellular senescence in kidney disease, particularly in diabetic kidney disease, the latest knowledge on senotherapy, and the potential and challenges of new therapeutic strategies, including combining extracellular vesicles and longevity proteins.

## Graphical abstract

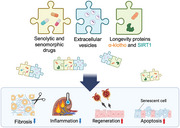

## 1 Cellular Senescence

**FIGURE 1:**
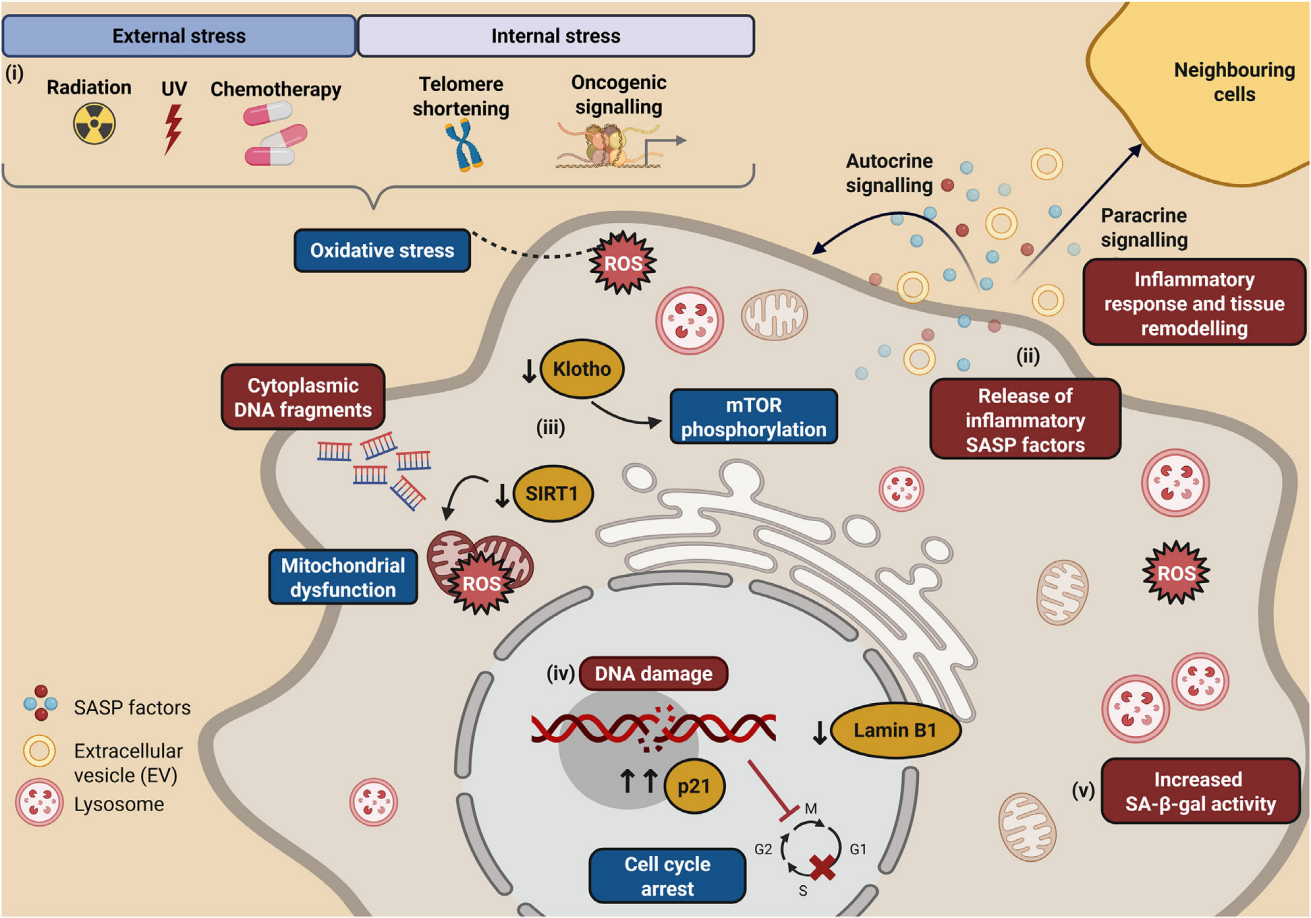
Potential causes and effects of cellular senescence. Major attributes of cellular senescence are summarized. (i) External factors that cause ageing associated‐cell cycle arrest include radiation, ultraviolet light, and chemotherapy. On the other hand, internal factors include the accumulation of reactive oxygen species (ROS), shortening of telomeres, and abnormal activation of oncogenes. (ii) The senescence‐associated secretory phenotype (SASP) is mainly regulated by the mammalian target of rapamycin (mTOR) pathway and the transcription factor nuclear factor kappa B (NF‐κB). SASP‐rich senescent cells propagate inflammatory interleukins, chemokines, growth factors, extracellular remodeling factors, and extracellular vesicles to neighboring cells (paracrine effect). Concurrently, these signals affect senescent cells themselves (autocrine effect). (iii) Decreased α‐klotho leads to mTOR phosphorylation and upregulation of SASP factors. A decrease in silencing information regulator 2‐related enzyme 1 (SIRT1), meanwhile, leads to abnormal accumulation of mitochondria, resulting in a decrease in energy production efficiency and an increase in ROS. (iv) DNA damage‐dependent and independent mechanisms modulate p53/p21Cip1, which plays an important role in initiating and maintaining cellular senescence, and irreversibly arresting the cell cycle. (v) The activity of β‐galactosidase (β‐gal), primarily present in lysosomes, is promoted by inflammatory cytokines.

Cellular senescence was first described by Hayflick and Moorhead in 1961 as the arrest of cell division in normal human cells after a certain number of divisions [1]. This phenomenon, termed replicative senescence, was later identified as an irreversible arrest of cell division induced by telomere shortening, with metabolic activity remaining intact [2, 3]. In addition to the replicative senescence, cellular senescence occurs due to stressors, such as oxidative stress, which arise from internal or external stressors (Figure 1). Internal stressors include oncogenes, such as rat sarcoma virus (Ras)^G12V^ and rapidly accelerated fibrosarcoma (Raf)^V600E^, while external stressors range from UV light, radiation, and chemicals, such as cigarette smoke [4, 5, 6, 7, 8, 9, 10]. Internal and external stressors work in unison to drive cellular senescence with DNA damage being a hallmark response.

At the molecular level, senescence is orchestrated by key signaling pathways, including the p53/p21^Cip1^ and p16^INK4a/Rb^ axes, which enforce cell cycle arrest. The mammalian target of rapamycin (mTOR) pathway and nuclear factor kappa B (NF‐κB) transcription factor are central regulators of the senescence‐associated secretory phenotype (SASP), a pro‐inflammatory secretome released by senescent cells [11, 12, 13, 14]. SASP factors include cytokines (e.g., interleukin/IL‐6, IL‐8, and tumor necrosis factor/TNF) [15, 16, 17], chemokines, growth factors (e.g., fibroblast growth factors/FGFs, vascular endothelial growth factors/VEGFs) [18, 19, 20], extracellular matrix remodeling enzymes, and extracellular vesicles (EVs) [21, 22]. These factors act in both autocrine and paracrine manners to reinforce senescence, propagate inflammation, and remodel tissue microenvironments [23, 24, 25]. While the SASP can promote chronic inflammation and tissue remodeling, cellular senescence itself also plays a beneficial role in tumor suppression and tissue homeostasis [26, 27]. Cellular senescence is thought to act as a cancer suppression mechanism that maintains genome homeostasis by arresting division of cells with damaged DNA that is challenging to repair [28, 29, 30]. Along with the arrest of cell division, the characteristics of cells that have undergone cellular senescence include cell enlargement, increased cytoplasmic DNA fragments [31], increased senescence‐associated‐beta‐galactosidase (SA‐β‐gal) [32], senescence‐associated heterochromatic foci (SAHF) [33], decreased lamin B1 [34], and resistance to apoptosis [35]. Senescent cells in tissues can be eliminated by SASP‐mediated immune cell mobilization, however, senescent cells accumulate in various tissues in the body as immune function declines with aging [36, 37]. Such accumulation could play a part in spurring age‐related diseases, including chronic kidney disease, atherosclerosis, chronic obstructive pulmonary disease, and cancer. Taken together, cellular senescence has a dual role: it serves as a cancer suppression mechanism by halting proliferation of damaged cells [29, 38], while the SASP can cause adverse events, such as tumor growth.

## 2 Cellular Senescence in Kidney Disease

Importantly, kidney aging does not occur in isolation. It both reflects and contributes to systemic aging [39, 40, 41]. The decline in kidney filtration, endocrine signaling, and metabolic homeostasis influences whole body aging processes [39, 41]. In addition, systemic aging factors, such as chronic inflammation, mitochondrial dysfunction, and impaired immune function further exacerbate kidney vulnerability [41, 42, 43]. The kidneys are metabolically active organs responsible for filtering approximately 47 gallons (about 180 L) of plasma ultrafiltrate per day, most of which is reabsorbed before excretion as urine [44]. This high metabolic demand makes them particularly susceptible to oxidative stress, inflammation, and structural damage over time. Unlike some other organs, the kidneys have a limited regenerative capacity, making age‐related functional decline largely irreversible. Aging leads to several structural and functional changes in the kidney, including a gradual decline in glomerular filtration rate, reduced renal blood flow, and cortical atrophy [45, 46]. These changes increase the susceptibility to chronic kidney disease and impair the kidney's ability to recover from acute injuries [47]. At the molecular level, kidney aging can be driven by a myriad of factors, including cellular senescence, inflammation, genomic instability, telomere attrition, mitochondrial dysfunction, impaired autophagy‐lysosomal function, and dysregulated signaling of longevity‐associated proteins such as silencing information regulator 2‐related enzyme 1/sirtuin‐1 (SIRT1) and α‑klotho [48]. Senescent cells accumulate in multiple kidney compartments, including glomerular epithelial cells, tubular epithelial cells, and endothelial cells. These cells secrete pro‐inflammatory cytokines and profibrotic factors as part of the SASP, exacerbating chronic inflammation and fibrosis, ultimately accelerating kidney aging [45]. Key molecular pathways implicated in this process include the p53/p21^Cip1^ and p16^INK4a/Rb^ axes, mTOR and NF‐κB, and persistent DNA damage signaling (Figure 1). The SASP amplifies chronic inflammation and fibrosis, further accelerating kidney aging and functional decline. Chronic kidney disease itself acts as an accelerator of biological aging, leading to premature aging and a disconnect between biological and chronological age [45]. As chronic kidney disease progresses, age‐related complications such as frailty become increasingly apparent [45, 47]. Understanding the molecular interplay between aging and kidney disease is, therefore, essential for developing strategies to mitigate premature organ aging and improve long‐term health outcomes. Diabetic kidney disease is a major complication of diabetes and a leading cause of chronic kidney disease, affecting approximately 30%–40% of diabetes patients worldwide [49]. Persistent hyperglycemia induces structural and functional alterations in the glomeruli, ultimately leading to kidney impairment. Notably, cellular senescence plays a significant role in diabetic kidney disease progression, as sustained hyperglycemia exacerbates oxidative stress and inflammatory responses, promoting the accumulation of senescent cells. These cells, particularly in the glomeruli and renal tubules, contribute to fibrosis and loss of kidney function, further accelerating disease progression. Importantly, experimental studies in murine diabetic kidney disease demonstrate that targeting senescence‑ and inflammation‑related pathways can ameliorate kidney injury and potentially influence systemic aging phenotypes. For example, antagonizing activin A signaling with follistatin reduces kidney fibrosis, injury, and senescence‑associated inflammation [50], while the senolytic combination dasatinib plus quercetin decreases kidney senescent cell burden and inflammation and restores geroprotective factors, such as α‑klotho and SIRT1 [51].The sections hereafter, review key molecular players involved in kidney disease, along with diagnostic and therapeutic approaches aimed at addressing premature kidney aging.

### 2.1 P21 Activation and Inflammation During Progression of Diabetic Kidney Disease

The activation of p21 (CDKN1A), a cyclin‐dependent kinase inhibitor associated with cellular senescence, is involved in the progression of diabetic kidney disease [52]. In hyperglycemic conditions, sustained activation of the p53/p21^Cip1^ pathway in renal proximal tubular cells leads to persistent cell cycle arrest, increased senescence, and enhanced production of pro‐inflammatory cytokines, such as TNF. This proinflammatory environment exacerbates kidney inflammation and promotes apoptosis, resulting in a progressive loss of functional renal cells [52, 53]. Notably, p21 is among the most persistently upregulated genes in response to chronic hyperglycemia, and its expression in both renal tubules and urine correlates with diabetic kidney disease severity, even after glycemic control is achieved [52, 53]. At the epigenetic level, persistent expression of p21 is associated with demethylation of its promoter and decreased DNA (cytosine‐5)‐methyltransferase 1 (Dnmt1) expression, contributing to persistent inflammation and sustained senescence [52]. In addition, hyperglycemia downregulates key longevity proteins, including SIRT1 and nicotinamide phosphoribosyltransferase [54, 55]. This reduction leads to increased p53 acetylation, further activating p21 transcription in proximal tubules [56]. These findings suggest that activation of SIRT1 may suppress p21 expression and reduce cellular senescence and tissue damage, making SIRT1 a promising therapeutic strategy. However, current therapies do not specifically target the persistent activation of the p53/p21^Cip1^ axis or the associated SASP, limiting their effectiveness in halting diabetic kidney disease progression. Furthermore, the resistance of senescent cells to apoptosis and their continued secretion of pro‐inflammatory and profibrotic factors emphasize the need for more targeted interventions.

### 2.2 Longevity Gene, Sirtuins, in Kidney Disease

SIRT1, a member of the sirtuin family of NAD+‐dependent deacetylases, is an evolutionarily conserved longevity gene with critical roles in cellular homeostasis from yeast to humans, and overexpression of SIRT1 has been reported to prolong lifespan in model organisms, such as yeast, *C. elegans*, and *Drosophila* [57, 58]. SIRT1 has a variety of anti‐aging effects, including protecting cells from oxidative stress, maintaining genomic stability, and regulating cellular metabolism, which collectively reduce the risk of age‐related diseases, such as diabetes, osteoporosis, and cancer [59, 60, 61, 62, 63, 64]. In the kidneys, SIRT1 expression declines with age and in disease states, including diabetic kidney disease [65, 66, 67]. Reduced SIRT1 activity leads to accumulation of dysfunctional mitochondria, impaired mitophagy and, increased oxidative stress in renal proximal tubular cells [68, 69, 70, 71]. Mechanistically, SIRT1 regulates mitochondrial quality control by deacetylating and activating transcription factors, such as PGC1α, FOXO, and p53, thereby, promoting mitochondrial biogenesis and autophagy while suppressing apoptosis and inflammation [72]. Moreover, a reduction in SIRT1 diminishes the expression of BCL2/adenovirus E1B 19 kDa interacting protein 3 (BNIP3) under hypoxic conditions. BNIP3 plays a central role in inducing autophagy in hypoxic environments, and the reduced expression of this protein impairs the activation of autophagy [73]. Consequently, clearance of abnormal mitochondria is impaired. The accumulation of abnormal mitochondria increases oxidative stress in renal proximal tubular cells [74]. Elevated oxidative stress enhances the mutation frequency of mitochondrial DNA, which is correlated with kidney dysfunction [75, 76, 77]. In diabetic states, SIRT1 expression in the proximal tubules is further suppressed, resulting in decreased secretion of nicotinamide mononucleotide (NMN), a key NAD+ precursor [78]. NMN supplementation in diabetic mouse models prevents disruption of the tubule‐glomerular junction and attenuates diabetic kidney disease progression, highlighting the therapeutic potential of targeting the SIRT1‐autophagy axis [79]. This finding suggests that activation of the SIRT1‐autophagy pathway may be leveraged as a potential therapeutic strategy to provide mitochondrial protection and reduce oxidative stress in aging kidneys. Additionally, activation of SIRT1 has been shown to suppress p21 expression, reduce cellular senescence, and attenuate tissue damage in preclinical models [80, 81, 82]. Consistent with these mechanistic insights, reduction, or genetic deletion of SIRT1 in mice aggravates aging‑induced organ injury and cellular senescence [83, 84]. Podocyte‑specific *Sirt1* knockdown mice exhibit accelerated aging‑associated glomerulosclerosis, albuminuria, and increased expression of senescence markers in glomeruli, suggesting that loss of SIRT1 promotes podocyte senescence in aging kidneys [83]. Heterozygous *Sirt1*‑deficient mice also show increased kidney apoptosis and fibrosis after injury, further supporting a protective, anti‑senescent role of SIRT1 in vivo [84]. However, SIRT1 activation alone may not fully reverse established fibrosis or resolve chronic inflammation due to the multifactorial nature of diabetic kidney disease pathogenesis.

### 2.3 Longevity Gene, α‐Klotho, in Kidney Disease

The transmembrane protein, α‐klotho, first identified as a key regulator of aging in 1997, when *Kl* (gene encoding for α‐klotho)‐deficient mice exhibited accelerated aging phenotypes, including markedly shortened lifespan, infertility, arteriosclerosis, skin atrophy, osteoporosis, and emphysema [85, 86]. α‐Klotho is predominantly expressed in the distal convoluted tubules of the kidney, as well as in the parathyroid gland, and exists in both membrane‐bound and soluble forms [85, 87, 88, 89]. In the kidney, α‐klotho functions as an obligate co‐receptor for fibroblast growth factor 23 (FGF23), enabling FGF23 signaling through fibroblast growth factor receptors. FGF23 is a phosphaturic hormone that plays a central role in regulating systemic phosphate homeostasis by reducing renal phosphate reabsorption and suppressing vitamin D activation [90, 91, 92]. In chronic kidney disease, *Kl* expression declines markedly, while FGF23 levels increase, particularly from chronic kidney disease stage 3 onward [76, 93, 94, 95, 96]. The dysregulation of the FGF23‐α‐klotho axis is associated with increased serum levels of inflammatory cytokines, including IL‐6 and TNF, and C‐reactive protein (CRP) [97]. Mechanistic studies demonstrate that reduced α‐klotho promotes kidney inflammation by enabling FGF23‐driven upregulation of pro‐inflammatory cytokines in renal tubular epithelial cells [97]. Furthermore, α‐klotho deficiency exacerbates transforming growth factor beta 1 (TGF‐β1)/mothers against decapentaplegic homolog 2 (SMAD2) and mammalian targets of rapamycin (mTOR) signaling, both of which are central to the development of kidney fibrosis and cellular senescence [98, 99, 100, 101, 102]. Recent research has also highlighted α‐klotho's role in modulating oxidative stress and cell death pathways. α‐Klotho suppresses NF‐κB activation, NLRP3 inflammasome assembly, and ROS production, thereby reducing podocyte pyroptosis and apoptosis in diabetic kidney disease models [103, 104]. Overexpression or supplementation of α‐klotho activates *Nrf2*, a master regulator of antioxidant responses, further protecting against high glucose‐induced oxidative stress and podocyte injury [98]. Collectively, these findings indicate that α‐klotho is a central regulator of kidney inflammation, fibrosis, and cellular senescence. The decline of α‐klotho in chronic kidney disease contributes to disease progression via activation of TGF‐β1/Smad2, mTOR, NF‐κB, and NLRP3 pathways, as well as impaired phosphate homeostasis. Restoring α‐klotho levels through recombinant protein administration, gene therapy, or pharmacological upregulation offers a promising therapeutic approach to suppress inflammation, reduce fibrosis, and slow the progression of chronic kidney disease [105, 106, 107]. However, further research is needed to optimize delivery, enhance kidney specificity, and clarify the long‐term benefits and risks of α‐klotho‐based interventions.

## 3 Small Molecules as Senotherapeutics

**FIGURE 2:**
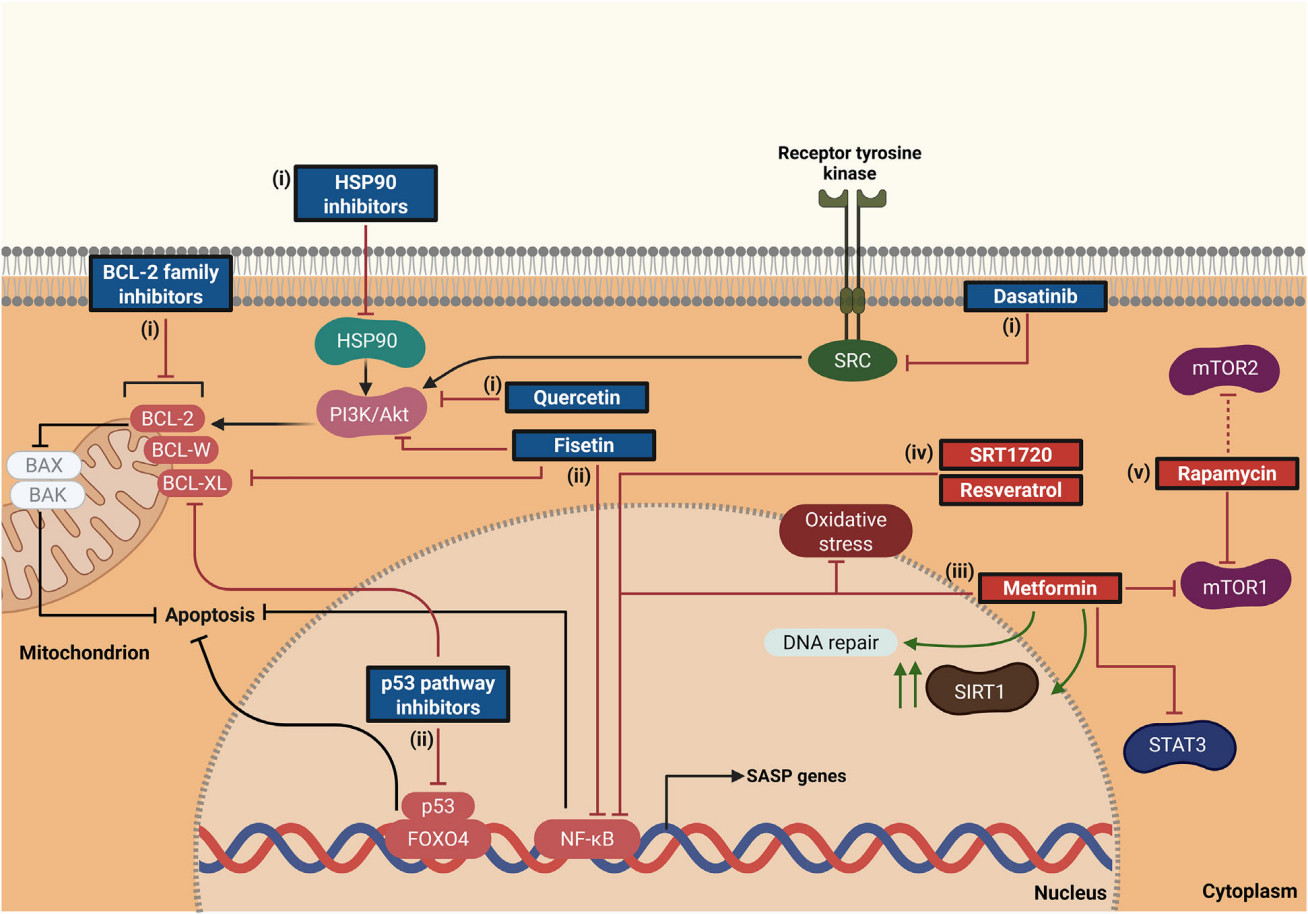
General representation of senotherapeutics and their action pathways. Senolytics induce apoptosis in senescent cells by disrupting pro‐survival mechanisms. (i) B‐cell lymphoma 2 (BCL‐2) inhibitors promote mitochondrial outer membrane permeabilization (MOMP) while heat shock protein 90 (HSP90) inhibitors, quercetin (phosphatidylinositol 3‐kinase/protein kinase B; PI3K/Akt), and dasatinib (proto‐oncogene tyrosine‐protein kinase; SRC) target upstream survival signaling. (ii) Nuclear pathways, including tumor protein 53/forkhead box protein O4 (p53/FOXO4) and NF‐κB, are disrupted by p53 inhibitors and fisetin, further sensitizing cells to apoptosis. Senomorphics, in contrast, modulate senescent cells without inducing apoptosis, primarily by suppressing SASP production. NF‐κB inhibition prevents SASP gene transcription, reducing inflammatory cytokines. (iii) Metformin inhibits NF‐κB, mTORC1, and signal transducer and activator of transcription 3 (STAT3) while promoting SIRT1 activation and DNA repair. (iv) SIRT1 activators (resveratrol, SRT1720) further suppress inflammation via NF‐κB. (v) Rapamycin exerts senomorphic effects by selectively inhibiting mTORC1, though off‐target effects on mTORC2 may occur.

As comprehensively reviewed in prior sections, minimizing the impact of senescent cells and SASP factors on an organism could potentially lead to safeguarding against a myriad of disease conditions, specifically age‐related ones. Senotherapeutic molecules are fundamentally classified into either of two groups, senolytics or senomorphics, where the former causes selective death of senescent cells, while the latter attenuates SASP factors to indirectly address senescence (Figure 2).

### 3.1 Senolytics

Although senescent cells contribute to pathological aging through SASP production and impaired repair capacity, they also play beneficial roles in tumor suppression, tissue homeostasis, wound healing, and regeneration. Therefore, therapeutic strategies that eliminate senescent cells or suppress SASP must be applied with caution to avoid disrupting these physiological functions. Additionally, intermittent dosing of these agents may provoke unanticipated effects, as discussed in subsequent sections. This dual nature of senescence highlights the need for careful evaluation of both the benefits and potential risks associated with senolytic and senomorphic interventions. Senescent cells evade apoptosis by upregulating anti‐apoptotic pathways, collectively termed senescent cell antiapoptotic pathways (SCAPs). Proteomic and transcriptomic analyses have revealed elevated expressions of SCAPs, fundamentally employed by senescent cells as deterrence against their own pro‐apoptotic SASP secretome [108]. In kidney disease contexts, senescent tubular epithelial and glomerular cells upregulate these SCAPs, contributing to kidney fibrosis and functional decline, making targeted senolytics particularly relevant for conditions like diabetic kidney disease (DKD) and acute kidney injury (AKI). Consistent with this, kidneys from patients with type 2 diabetic nephropathy exhibit an accelerated senescent phenotype with increased SA‐β‐gal and p16^INK4A^ in tubular cells and podocytes and shortened telomeres, supporting cellular senescence in the kidney as an early pathogenic driver in diabetic nephropathy [109]. Experimental work further shows that senescent proximal tubular epithelial cells impair post‐injury regeneration and promote fibrosis, while pharmacological clearance of these cells in aged or irradiated mouse kidneys restores tubular proliferation, improves function, and reduces fibrosis, underscoring senolytics as a promising strategy to protect vulnerable kidneys [110, 111, 112]. Accordingly, bioinformatic approaches led to the identification of the first senolytic compounds, which mechanisms of action involve ablating senescent cells by targeting imperative SCAP network nodes involved in pro‐survival mechanisms [108]. These include the tyrosine kinase inhibitor, dasatinib, as well as naturally occurring flavonoids like quercetin and fisetin. Substantial heterogeneity exists in SCAP nodes across varying senescent cell populations, relying on a myriad of players: ephrin receptors, B‐cell lymphoma 2 (BCL‐2) family members, and phosphatidylinositol 3‐kinase (PI3K)/protein kinase B (Akt) [108, 113]. This heterogeneity can contribute to senolytics preferentially removing specific subpopulations of senescent cells. Additionally, efforts to reduce off‐target effects on non‐senescent cells and the “panolytic” nature of several of these candidates have led to high‐throughput library screens and the inception of second‐generation senolytics that increase specificity for senescent cells [114, 115].

#### 3.1.1 Dasatinib and Quercetin

Dasatinib and quercetin were discovered through a hypothesis‐driven bioinformatic deduction [108]. Dasatinib, a United States’ Food and Drug Administration (FDA)‐approved anti‐cancer drug, was shown to inhibit cellular proliferation and migration while inducing apoptosis, by virtue of being an ephrin receptor inhibitor [108]. Since ephrins facilitate an integral pro‐survival pathway in senescent cells, the administration of dasatinib in preclinical studies reduced the viability of these cells and ameliorated ageing phenotypes both in vitro and in vivo [108]. Meanwhile, quercetin, a polyphenol with potent antioxidant properties, is involved in influencing diverse signaling cascades, specifically demonstrating senolytic capacity both in vitro and in vivo via inhibiting PI3K/Akt and p53/p21/serpine pathways (Table 1) [108, 116]. A follow‐up study assessing the synergistic effect of dasatinib and quercetin was shown to be more effective in preclinical models in inducing apoptosis and targeting more SCAP network nodes in comparison to either drug alone in several senescent cell subpopulations [108]. This enhanced efficacy led to improvements in several age‐associated diseases and disorders in mice including metabolic dysfunction, hepatic steatosis, and pulmonary fibrosis, to name a few [117, 118, 119]. In kidney‐specific models, dasatinib in combination with quercetin has shown promise in attenuating DKD progression by reducing glomerular senescence, podocyte injury, and kidney fibrosis, though it exacerbated injury markers in experimental AKI, highlighting context‐dependent effects [120, 121]. In streptozotocin‐induced murine DKD, a short “hit‐and‐run” D+Q regimen improved kidney function, reduced tubular and glomerular injury, senescence and inflammation markers, and restored geroprotective factors such as α‐klotho and SIRT1, reinforcing its potential as a kidney‐targeted senolytic strategy [51]. Meanwhile, in an open‐label phase 1 pilot study, a 3‐day oral administration of the aforementioned senolytic combination effectively reduced adipose tissue senescent cell burden in humans, suggesting translational potential [122]. The senolytic combination is also being studied in several clinical trials for various pathologies, with skeletal health (NCT04313634) and Alzheimer's disease (NCT05422885) to name a few. However, it is worth highlighting that the efficacy of dasatinib and quercetin is cell‐type dependent, and dasatinib carries risks of myelosuppression (neutropenia, thrombocytopenia, anemia) and pleural/pericardial effusions (5%–15% annual incidence in CML patients, sometimes requiring discontinuation) [83, 92]. Although short, intermittent combination courses of dasatinib and quercetin (e.g., aforementioned 3‐day “hit‐and‐run” regimens) have been generally well tolerated in small early‐phase senolytic trials with no serious drug‐related adverse events, these studies involved limited sample sizes (*n* < 20) and brief follow‐up periods, leaving long‐term safety, cumulative toxicity risk, and optimal intermittent dosing schedules in humans undetermined [123, 124].

**TABLE 1:** Comparison of senotherapeutic strategies for kidney disease.

Therapeutic approach	Mechanism of action	Advantages/effects	Disadvantages	Refs.
Senolytics				
Dasatinib	SRC family kinase inhibitor	↓ Anti‐apoptotic signaling ↑ apoptosis	Combination therapy demonstrates cell type specific senolytic‐activity, limiting broad effectiveness	[108]
Quercetin	PI3K/Akt inhibitor p53/p21/serpine inhibitor	↓ Anti‐apoptotic signaling ↑ apoptosis		[108, 116]
Fisetin	↓ Anti‐apoptotic Bcl‐2 proteins NF‐κB pathway inhibitor PI3K/Akt inhibitor	↑ Apoptosis	Cell type‐specific senolytic activity, limiting broad effectiveness	[126, 127, 130]
*BCL‐2 family member inhibitors*				
ABT‐263 (Navitoclax)	BCL‐2, BCL‐W and BCL‐xL inhibitor	↑ Mitochondria‐mediated apoptosis (caspase cascade)	Cell type‐specific senolytic activity, limiting broad effectiveness; risk of thrombocytopenia and neutropenia	[132, 133, 134]
*HSP90 inhibitors*				
Geldanamycin	HSP90 signaling inhibitor PI3K/Akt inhibitor	↓ Anti‐apoptotic signaling ↓ SASP production ↑ mitochondria‐mediated apoptosis (caspase cascade)	Off‐target toxicity (non‐senescence cells); treatment resistance due to induction of compensatory pathways (HSP70)	[114, 135]
17‐AAG (tanespimycin)				
17‐DMAG (alvespimycin)				
*p53 pathway inhibitors*				
FOXO4‐DRI	FOXO4/p53 inhibitor	p53 nuclear exclusion ↑ mitochondria‐mediated apoptosis (caspase cascade)	Off‐target toxicity (non‐senescence cells)	[142]
Senomorphics				
Rapamycin	mTORC1 inhibitor	↓ Anti‐apoptotic signaling ↓ SASP production ↓ oxidative stress	Off‐target inhibitor of mTORC2, leading to metabolic dysregulation, thrombocytopenia, hyperlipidaemia, and impaired wound healing	[154, 160]
Metformin	IκB inhibitor ↓ NF‐κB ↑ Nrf‐2 mediated glutathione peroxidase 7 ↓ STAT3 ↑ AMPKα1 ↑ SIRT1 ↓ mTORC1	↓ SASP production ↓ oxidative stress ↑ DNA repair	Lactic acidosis	[151, 163, 164, 165, 167]
Resveratrol	↑ SIRT1 IκB inhibitor NF‐κB pathway inhibitor	↓ SASP production ↓ oxidative stress	Biphasic dose‐dependent effects: pro‐oxidant and induces senescence at higher concentrations; diet‐based variability in in vivo models	[171, 173, 174, 175]
Sirtuin‐activating compounds	↑ SIRT1 IκB inhibitor NF‐κB pathway inhibitor	∼1000‐fold greater potency than resveratrol in vitro (SIRT1 activation); robust lifespan extension in high‐fat diet‐fed mice, with more modest and variable effects under standard diet conditions	Possibility of affecting immune‐related pathways in non‐aging cells and tissues, causing abnormal regulation of tissue function and side effects	[176, 177, 178, 179]
Longevity proteins				
α‐Klotho	↓ Wnt1 and Wnt9a‐induced mitochondrial damage	↓ Premature kidney tubular cell aging ↓ senescence	Risk of excessive Wnt signal inhibition	[101]
SIRT1	Deacetylating p53, FOXO, (RelA)/p65NF‐κB, STAT3, and (PGC1α)/(PPARγ)	↓ Podocyte injury ↓ senescence ↓ Diabetic kidney disease progression	No uniform therapeutic effects due to disease‐specific variation in signaling pathways (p53, FOXO, NF‐κB, STAT3, PGC1α/PPARγ); SIRT1 activation may suppress immune function by reducing inflammatory cytokine expression, increasing infection risk; incomplete suppression of kidney fibrosis	[188, 190]
Nicotinamide	Smad and STAT pathway inhibition	↓ Kidney fibrosis ↓ inflammatory markers	SIRT1 dependent effects; potential immunosuppressive effects	[190]
Stem cell‐based therapy				
MSCs	SASP factors including EVs ↓ TGF‐β/Smad pathway	↓ Oxidative stress ↓ senescence ↓ fibrosis ↑ angiogenesis ↑ extracellular matrix remodeling	Potential tumorigenicity and vascular obstructions; complex manufacturing, handling, and storage	[200, 201]
MSC EVs	NLRP3 pathway inhibitor NF‐κB pathway inhibitor ↓ KLF6 ↑ M2 macrophage polarization Regulate p53‐p21 axis	Cell‐free strategy ↓ senescence ↓ inflammation (IL‐6, IL‐1β, IL‐18, and TNF) ↓ Oxidative stress Improves nuclear structure and function	Partial therapeutic effects	[193, 228, 229, 230, 234, 237, 271, 272]

Abbreviations: AMPKα1, adenosine monophosphate‐activated protein kinase; Wnt, wingless‐related integration site; RelA, v‐rel avian reticuloendotheliosis viral oncogene homolog A; PGC1α, peroxisome proliferator‐activated receptor‐γ coactivator 1‐α; PPARγ, peroxisome proliferator‐activated receptor gamma; TGF‐β1, transforming growth factor beta 1; KLF6, kruppel‐like factor 6; NLRP3, nucleotide‐binding domain‐like receptor 3; TNF, tumor necrosis factor.

#### 3.1.2 Fisetin

Similar to quercetin but more potent, fisetin is another natural flavonoid engaged in a myriad of signaling pathways [125]. Fisetin appears to function by downregulating nuclear factor‐kappa B (NF‐κB) and anti‐apoptotic BCL‐2 family proteins, and modulating the PI3K/Akt/mTOR pathway (Table 1) [126, 127]. Recent studies have highlighted fisetin's therapeutic potential in mitigating senescence‐associated alterations in the kidney, with murine models of chronic kidney disease, demonstrating ameliorated senescent cell burden and kidney fibrosis, with improved tubular function observed in preclinical models [128, 129]. While fisetin demonstrates low toxicity even at high dosages, its potential as a broad senolytic is limited by its cell‐specific activity, necessitating further exploration for pan‐cellular senolytics [130].

#### 3.1.3 BCL‐2 Family Member Inhibitors

Senescent cells resist apoptosis largely through upregulation of anti‐apoptotic BCL‐2 family proteins, including BCL‐2, Bcl‐extra large (BCL‐xL), and Bcl‐2‐like protein 2 (BCL‐W). These proteins inhibit mitochondrial outer membrane permeabilization (MOMP), a key step in apoptosis, by binding to and sequestering pro‐apoptotic proteins, such as Bcl‐2‐associated X protein (BAX) and Bcl‐2 antagonist/killer (BAK), impeding their oligomerization and pore formation [131]. BH3 mimetics such as ABT‐263 (Navitoclax), ABT‐737, A‐1331852, and A‐1155463 were developed to overcome this resistance [130]. These small molecules competitively bind to the BH3‐biding groove of BCL‐2 family proteins, displacing BAX and BAK, and reactivating the apoptotic cascade. Navitoclax is a BH3 mimetic that inhibits the anti‐apoptotic proteins BCL‐2, BCL‐xL, and BCL‐W, which are required for the survival of certain senescent cell populations according to RNA interference studies (Table 1) [132]. In vitro studies validated the senolytic potential of Navitoclax, specifically in senescent human umbilical vein endothelial cells (HUVECs), IMR90 human lung fibroblasts, and murine embryonic fibroblasts, while human primary preadipocytes remained unaffected [133]. In euglycemic conditions, Navitoclax has shown selective elimination, in preclinical models, of senescent cells within the proximal tubular epithelium of both young and aged mice with acute kidney injury, with improved renal function observed [112]. However, the efficacy of BCL‐2 family inhibitors is highly dependent on the expression profile of anti‐apoptotic proteins in different senescent cell types, leading to incomplete clearance of all senescent populations. It is important to note that Navitoclax can cause adverse clinical effects, including thrombocytopenia and neutropenia, plausibly attributed to the inhibition of BCL‐2 family members in an indiscriminate manner [134]. This off‐target toxicity limits their clinical applicability, especially for chronic conditions like kidney disease, where long‐term safety is paramount.

#### 3.1.4 HSP90 Inhibitors

A distinct class of senolytic drug candidates are heat shock protein (HSP) 90 inhibitors, which demonstrate promising senotherapeutic activities in a pan‐cellular manner. HSP90 is a molecular chaperone essential for the stabilization and function of many survival‐related proteins, including those in the PI3K/Akt/mTOR pathway (Table 1) [114, 135]. Senescent cells depend on HSP90 to sustain anti‐apoptotic signaling and avoid programmed cell death. HSP90 inhibitors are thought to act similar to flavonoids, such as quercetin and fisetin, by suppressing the PI3K/Akt/mTOR signaling cascade [135]. Compounds such as geldanamycin, 17‐AAG (tanespimycin), and 17‐DMAG (alvespimycin) were identified through a medium‐throughput screening platform based on a SA‐β‐gal assay. These agents have demonstrated senolytic activity in vitro in various cell types, including mouse and human fibroblasts, mesenchymal stem cells, and vascular endothelial cells, regardless of the senescence‐inducing stimulus such as oxidative, genotoxic, or replicative stress [114]. In vivo, intermittent administration of 17‐DMAG in progeroid mouse models reduced renal p16^INK4a^ expression, delayed onset of multiple age‐related symptoms, and prolonged health span [114]. However, HSP90 inhibitors are not uniformly effective across all cell types and can induce off‐target toxicity due to their broad inhibition profile [136, 137, 138]. Notably, compensatory upregulation of other heat shock proteins such as HSP70 may lead to treatment resistance. While HSP90 inhibitors offer broad senolytic potential and have shown efficacy in both preclinical in vitro and in vivo models, their clinical translation is challenged by cell‐type specificity, off‐target toxicity, and the risk of resistance. Additionally, they exhibit narrow therapeutic windows due to their central role in proteostasis, causing dose‐limiting toxicities including hepatotoxicity (transaminase elevations), gastrointestinal intolerance, fatigue, and ocular adverse events (blurred vision, keratitis) in oncology trials, which have constrained their clinical development as systemic agents [138, 139, 140]. Future strategies should focus on improving selectivity, minimizing adverse effects, and exploring rational combination therapies to maximize their therapeutic benefit in aging‐related diseases such as chronic kidney disease.

#### 3.1.5 p53 Pathway Inhibitors

Transcription factor p53 is a central regulator of DNA repair, apoptosis, and the initiation and maintenance of cellular senescence. Recently, p53 and its regulatory networks have emerged as promising targets for senolytic development. For instance, by binding to p53 at sites of DNA damage during irradiation‐induced senescence, and inhibiting its nuclear export, forkhead box protein O4 (FOXO4) proteins upregulate the expression of p21, thus, maintaining and promoting a p16‐independent senescence response [141]. To disrupt this interaction, the peptide FOXO4‐DRI was developed to interfere with FOXO4/p53 binding, resulting in the nuclear exclusion of p53, and subsequent binding to BCL‐xL and activation of mitochondria‐mediated apoptosis via the caspase cascade (Table 1) [142].

Despite encouraging findings, p53 pathway inhibitors and other senolytics face substantial hurdles to clinical translation. Many of these agents were originally developed for cancer or unrelated conditions and have since been repurposed for senolytic applications, potentiating several off‐target effects downstream due to limited specificity [143, 144, 145]. For instance, dasatinib can induce myelosuppression and cardiac events, particularly in older adults or individuals with comorbidities, while flavonoids like quercetin exert broader biological effects, including modulation of oxidative stress and interference with autophagy. Additionally, tissue atrophy may occur because of the abrupt ablation of senescent cells by senolytics, given that senescent cells impart considerable structural integrity to aged tissues [146, 147]. The heterogeneity of senescent cell populations and their reliance on diverse survival pathways further complicate the development of universally effective senolytics, emphasizing the need for more refined approaches. It is also important to recognize that senescent cells play beneficial roles, contributing to wound healing, cellular reprogramming, and tissue regeneration [148, 149, 150]. Consequently, it is vital to reevaluate and enhance the application of current senolytics, either by (1) increasing their target specificity, or (2) addressing another critical feature of senescent cells, SASP factors, to mitigate the deleterious effects of senescence. This has sparked increasing interest in targeting the SASP through senomorphic agents, which aim to attenuate the harmful paracrine effects of senescent cells while preserving their beneficial roles.

### 3.2 Senomorphics

An alternative approach to senolytics, are senomorphics that indirectly target cellular senescence by attenuating pathological SASP factors without causing cell death. As highlighted earlier, senescent cells are characterized by the accretion of a pro‐inflammatory proteolytic secretome known as SASP, featuring a complex blend of signaling molecules. These signaling molecules cumulatively augment and reinforce senescence via autocrine and paracrine mechanisms. Such processes are primarily stimulated by the activation of NF‐κB and mTOR [13, 151], as well as cascades such as p38 mitogen‐activated protein kinases (p38MAPK) signaling [152]. As a result, targeting these pathways, and thus suppressing SASP, could possibly be the preferred method of mitigating the effects of senescence, while sparing senescent cells that are otherwise implicated in advantageous processes like wound healing and growth.

#### 3.2.1 Rapamycin

A macrolide compound isolated from the bacterium *Streptomyces hygroscopicus*, rapamycin is recognized as one of the most well‐established senomorphics, suppressing senescence and SASP factors across various senescent mouse, rat, and human cells [153]. Rapamycin primarily exerts effects by modulating the mTOR signaling pathway, which orchestrates crucial processes such as cell growth, proliferation, aging regulation, and cellular senescence. Of the two distinct multiprotein complexes that centrally involve mTOR, TOR‐complex 1 (mTORC1), and mTORC2, the senomorphic effects of rapamycin are primarily attributed to relatively selective inhibition of mTORC1 (Table 1) [154]. Numerous in vivo studies have shown that rapamycin can reduce age‐related dysfunction, delay tumor onset and ageing, and extend lifespan [155, 156, 157]. In renal in vivo models, rapamycin inhibits mTORC1 to suppress SASP‐driven tubulointerstitial fibrosis, protects kidney allografts from preservation/reperfusion‐induced senescence, and delays DKD progression by enhancing autophagy and reducing p21 expression [158]. However, although rapamycin primarily targets mTORC1, chronic administration can also disrupt mTORC2 assembly in certain tissues, impairing critical signaling pathways involved in metabolic homeostasis and cytoskeletal organization, to name a few [159]. As a result, off‐target inhibition of mTORC2 has been directly associated with adverse side effects, such as metabolic dysregulation, thrombocytopenia, hyperlipidaemia, and impaired wound healing, prompting the development of novel analogues with enhanced target specificity, improved potency, and more favorable pharmacokinetic profiles [160].

#### 3.2.2 Metformin

Recently, the antidiabetic drug, metformin, has received attention due to anti‐aging properties, sparking interest in its potential repurposing as a senotherapeutic. Metformin has demonstrated geroprotective effects by reducing inflammation and senescence markers in in vitro studies with human mesenchymal stem cells, while also extending lifespan and delaying age‐related metabolic decline in rodent models [161]. Building on these findings, the Targeting Aging with Metformin (TAME) trial is a landmark, ongoing multicenter randomized controlled study enrolling 3000 non‐diabetic older adults to directly test whether metformin can delay the onset of multiple age‐related diseases, including cardiovascular events, cancer, dementia, and mortality, thereby, establishing its potential as a geroprotective intervention in humans [162]. However, despite these benefits, the underlying mechanisms of metformin remain complex and not fully understood. Metformin is proposed to act as a pleiotropic agent, modulating multiple pathways and molecular targets. For instance, metformin is known to inhibit phosphorylation of two catalytic subunits of I‐kappa‐B (IκB) kinase, an upstream activator of NF‐κB, thereby, suppressing SASP production (Table 1) [151]. Additional mechanisms proposed include the upregulation of nuclear factor erythroid 2‐related factor 2 (Nrf2)‐mediated glutathione peroxidase 7 and downregulation of the signal transducer and activator of transcription 3 (STAT3) pathway [163, 164]. Additionally, metformin's therapeutic potential in age‐related diseases may be attributed to its influence on several hallmarks of aging, either directly or indirectly, activating the alpha‐1 catalytic subunit of the 5’‐adenosine monophosphate‐activated protein kinase (AMPKα1) and SIRT1, downregulating insulin and mTORC1, reducing oxidative stress, and enhancing DNA repair, among others [165]. In a rat model of non‐diabetic chronic kidney disease, metformin exerted renoprotective properties and ameliorated senescence, as evidenced in proteomic analysis [166]. However, the fundamental limitation of using metformin as a senomorphic in chronic kidney disease patients is the associated risk of lactic acidosis, due to impaired renal clearance, resulting in decreased clearance and accumulation of metformin in patients with impaired renal function. Even with dose adjustments in the case of advanced disease, the potential severity of metformin still curtails its use in moderate to severe chronic kidney disease cases as a senotherapeutic, particularly when the estimated glomerular filtration rate falls below 30 mL/min/1.73 m^2^ [167, 168].

#### 3.2.3 Resveratrol

SIRT1, a central modulator of multiple signaling and transcriptional pathways involved in senescence and aging, shows reduced expression with age; however, its upregulation effectively mitigates cellular senescence and extends lifespan in several organisms [67, 169, 170]. Resveratrol has been explored as a potential SIRT1 activator, and hence, a senomorphic that could alleviate the effects of senescence and more specifically, SASPs. Resveratrol exhibits a complex biological profile by modulating multiple pathways, including NF‐κB, where it acts as an IκB inhibitor, similar to metformin. Resveratrol enhances SIRT1 activity and attenuates NF‐κB signaling primarily by inhibiting IKK‐dependent phosphorylation of IκB. Consequently, IκB degradation is reduced, NF‐κB nuclear translocation and transcriptional activity are suppressed, and SASP mediators, such as IL‐6 and IL‐8 are downregulated in senescent or aging tissues [171]. Functionally, this positions resveratrol as a senomorphic agent that modulates NF‐κB driven secretory programs and mitigates inflammaging without directly eliminating senescent cells, as observed with metformin [171]. Impediment of high glucose‐induced kidney cell senescence has been demonstrated with resveratrol, and in aged mice, protection against glomerulosclerosis occurs via SIRT1‐mediated *Kl* expression [172]. Concurrently, resveratrol exhibits a biphasic dose‐dependent effect on senescence, functioning as a pro‐oxidant and inducing senescence in multiple cell lines at higher concentrations (Table 1) [173, 174]. Additionally, the geroprotective effects of resveratrol in mice are more nuanced, with significant lifespan extension observed primarily in those fed a high‐fat diet, but not in mice on a standard diet [175]. This variability motivates the development of advanced sirtuin‐activating compounds with enhanced bioavailability, stability, and potency. While SRT1720 has been shown to substantially extend lifespan in high‐fat diet‐fed mice, lifespan extension under standard diet conditions is more modest and less consistent across studies, and should therefore be interpreted with caution (Table 1) [176, 177]. SRT2104 has substantial tolerability and bioavailability in humans, progressing into numerous clinical trials for age‐related diseases [178, 179].

Senomorphics provide an indirect method to target senescence, which potentially necessitates continued treatment since senescent cells can resume detrimental activity upon cessation of therapy [180, 181]. Additionally, systemic administration of senomorphics inadvertently interferes with several immune‐related pathways in non‐senescent cells and tissues, which could dysregulate tissue function and lead to off target effects [180, 182, 183]. These shortcomings may impede the translation of senomorphics into clinically approved drugs.

Taken together, small molecule senotherapeutics such as senolytics and senomorphics present a promising class of drugs to treat senescence observed in a several pathologies, including chronic kidney disease. However, other therapeutic agents based on macromolecules, such as proteins, are emerging as complementary strategies to address senescence in aging.

## 4 Longevity Proteins as Therapeutic Agents

The accumulation of senescent cells leads to a self‐perpetuating cycle of damage and fibrosis, through multiple pathways triggered by SASP factors and iron accumulation. Tissue fibrosis is exacerbated by persistent accumulation of iron within senescent cells, even after extracellular iron depletion, which fuels the generation of reactive oxygen species and further promote SASP production [184]. A sizeable population of SASPs include pro‐fibrotic factors such as serine proteinase inhibitor family E member 1 (SERPINE1) and IL‐11 [185, 186], as well as inflammatory soluble biomolecules that attract immune cells to the tissue locality to perpetuate inflammation. Fibrosis progresses as healthy parenchyma is replaced by collagen‐rich scar tissue, leading to disruption of normal organ architecture and loss of function [118, 187]. Consequently, it is essential to address not only the primary manifestations of senescence by modulating senescent cell populations and SASP factor secretion, but also the downstream consequences on tissue function, particularly fibrosis and the progressive decline in organ functionality. Longevity proteins, including α‐klotho and SIRT1, offer targeted strategies to counteract these processes by modulating anti‐aging and anti‐fibrotic signaling pathways.

For example, α‐klotho inhibits Wnt/β‐catenin and TGF‐β/Smad signaling, suppresses oxidative stress, and regulates phosphate metabolism. Recombinant human α‐klotho supplementation has been shown to ameliorate kidney fibrosis in vitro through inhibition of wingless‐related integration site Wnt1‐ and Wnt9a‐induced mitochondrial damage, thereby preventing premature senescence of kidney tubular cells (Table 1) [101]. In vivo, α‐klotho markedly reduced fibrotic lesions in unilateral ischemia‐reperfusion mice by modulating Wnt/β‐catenin signaling, underscoring the therapeutic potential of α‐klotho in kidney diseases [101]. Importantly, recent advances in gene therapy have enabled the targeted delivery of the *Kl* gene, encoding α‐klotho, to injured renal tubular epithelial cells using nanoparticles [107]. In a 2024 study, Zhang et al. demonstrated that polydopamine‐polyethylenimine‐L‐serine‐*Kl* plasmid nanoparticles (PPSK NPs) can safely and selectively deliver *Kl* to damaged kidney cells, restore α‐klotho expression, and effectively prevent the progression from acute kidney injury to chronic kidney disease in mouse models [107]. The protective effect of this nanoparticle‐based therapy was shown to be mediated by upregulation of PPARα and improved fatty acid β‐oxidation, resulting in reduced kidney lipid accumulation and fibrosis. In addition to α‐klotho, SIRT1 is another well‐studied longevity protein with significant renoprotective and anti‐fibrotic properties. Recent research has highlighted the pivotal role of SIRT1 in maintaining kidney homeostasis and counteracting cellular senescence and fibrosis, particularly in the context of diabetic kidney disease.

Several aforementioned senotherapeutics, namely metformin and resveratrol, have demonstrated restorative actions on SIRT1. SIRT1 is believed to mediate its renoprotective effects in diabetic kidney disease by deacetylating transcription factors involved in disease pathogenesis, including p53, FOXO, v‐rel avian reticuloendotheliosis viral oncogene homolog A (RelA)/p65NF‐κB, STAT3, and peroxisome proliferator‐activated receptor‐γ coactivator 1‐α (PGC1α)/peroxisome proliferator‐activated receptor gamma (PPARγ) (Table 1) [188]. Podocyte‐specific overexpression of SIRT1 in type 1 diabetic OVE26 mice alleviates podocyte injury and slows disease progression [189]. Mechanistically, SIRT1 activation suppresses pro‐senescent and pro‐fibrotic gene expression, while enhancing mitochondrial biogenesis and autophagy. Pharmacological activation of SIRT1 can be achieved by supplementation with nicotinamide adenine dinucleotide (NAD+) precursors, such as nicotinamide. Systemic nicotinamide administration in mice reduces kidney fibrosis by inhibiting phosphorylation of Smad and STAT pathway components, with in vitro data supporting its immunosuppressive and anti‐fibrotic effects [190]. The specificity of this effect is confirmed by reversal with the SIRT1 inhibitor EX527, demonstrating that nicotinamide renoprotective actions are at least partly SIRT1‐dependent [190].

While longevity proteins, such as α‐klotho and SIRT1, as well as their pharmacological activators, have shown promise in modulating key anti‐aging and anti‐fibrotic pathways in preclinical models, their clinical translation remains limited by challenges related to delivery, specificity, and the multifactorial nature of kidney aging. These limitations have prompted the search for alternative or complementary therapeutic strategies that more broadly target the complex microenvironment of the aging kidney. In this context, mesenchymal stem cells (MSCs) and their EVs have emerged as promising candidates due to their multifaceted immunomodulatory and reparative properties.

## 5 MSCs and EVs as Therapeutic Agents

Several small molecule senomorphics with potential to target senescence‐associated inflammation have been explored. However, as previously mentioned, many have limitations, that impede their clinical use, including the risk of off‐target effects in non‐senescent cells. Given these challenges, an alternative approach is to leverage MSCs, which possess comprehensive anti‐inflammatory and repair properties with favorable safety profiles [191, 192].

MSCs are a type of adult stem cell with the ability to self‐renew and differentiate into multiple cell types. MSCs can be isolated from a variety of tissues, including adipose tissue, placenta, bone marrow, and umbilical cord [193, 194]. Often referred to as “injury drugstores,” MSCs exhibit immunomodulatory, regenerative, and anti‐inflammatory properties across a range of in vitro and in vivo models, making them promising as a treatment for various diseases, including chronic kidney disease [195]. In rodent models, MSCs have consistently demonstrated kidney recovery through various mechanisms, including producing a secretome rich in biomolecules that ameliorate oxidative stress, senescence, and fibrosis, promote angiogenesis, and induce extracellular matrix remodeling [196]. MSC‐based therapies have already entered clinical practice, with Ryoncil (remestemcel‐L‐rknd) becoming the first FDA‐approved MSC therapy for treating steroid‐refractory acute graft‐versus‐host disease (SR‐aGVHD) in pediatric patients as young as 2 months [197, 198]. This milestone highlights the growing potential of MSCs in translational medicine. In fact, a placebo‐controlled, randomized phase 1b/2a clinical trial assessing bone marrow‐derived anti‐CD362–selected, allogeneic MSCs in adults with progressive diabetic kidney disease (ORBCEL‐M) observed reduced estimated glomerular filtration rate deterioration, in conjunction with favorable safety and tolerability outcomes [199]. There are also several other early‐phase MSC clinical trials in chronic kidney disease, including NCT06752577 and NCT04869761.

While MSC‐based therapies have shown promise, their clinical application is limited by concerns, such as, tumorigenicity, vascular obstruction, and poor engraftment efficiency (Table 1) [200, 201]. Studies have also revealed that less than 1% of transplanted MSCs successfully reach the target tissue, with the majority getting trapped in organs, such as the liver, spleen, and lungs [202, 203]. These limitations have led to growing interest in cell‐free strategies, especially the use of EVs derived from MSC secretomes, which are thought to mediate many of the beneficial paracrine effects of MSCs [204, 205]. EVs are nano and microscale membrane‐encapsulated carriers produced via various membranous pathways in response to extracellular cues [206]. EVs are much smaller than cells and can display improved distribution throughout the body through ease of transport in the blood and lymphatic systems [207]. EVs carry biomolecules such as lipids, proteins, carbohydrates, and RNAs, that modulate cellular function at proximal and distal sites [208, 209, 210, 211, 212]. Multiple preclinical studies have demonstrated that MSC‐derived EVs reduce inflammation and fibrosis in rodent models of diabetic nephropathy, ischemia‐reperfusion injury, and chronic kidney disease by modulating specific pathways such as NLRP3 and toll‐like receptor 4 (TLR4) [204, 213, 214, 215, 216, 217, 218, 219, 220]. MSC EVs have also progressed to clinical trials, including a phase 3 trial for acute respiratory distress syndrome [221]. It is worth noting that in addition to MSCs, other EV sources also show promise in treating kidney disease, including clinical‐grade EV products derived from platelets [222] and HEK293 human embryonic kidney cells [223].

Previous studies demonstrated accumulation of intravenously injected MSC EVs in the injured kidney of mice with renal ischemia‐reperfusion injury [224, 225]. MSC EVs alleviate acute kidney injury by inhibiting apoptosis, inflammation, and oxidation, and regulating angiogenesis, the cell cycle, regeneration, autophagy, and proliferation [226]. In a recent study, Wang and colleagues showed that MSC EVs inhibit inflammation, including the expression of IL‐6, IL‐1β, IL‐18, and TNF, in a mouse model of diabetic nephropathy [193]. The EVs suppressed the nucleotide‐binding domain‐like receptor 3 (NLRP3) signaling pathway, which plays an important role in inflammatory responses. Another study demonstrated that MSC EVs protected podocytes and diabetic mice from inflammation by mediating the NLRP3 inflammasome [227]. The anti‐inflammatory effects were primarily attributed to miR‐22‐3p in the EVs, as knockdown of this miR, which targets the NLRP3 inflammasome signaling pathway, resulted in a loss of anti‐inflammatory effects in vitro and in vivo [227]. Moreover, MSC EVs with NRF2 mitigated oxidative stress by reducing reactive oxygen species (ROS) levels and suppressing pro‐inflammatory cytokines, including IL‐1β, IL‐6, and TNF, under high‐glucose conditions (Table 1) [228].

Other miRs, such as miR‐146a and miR‐10a‐5p, are also enriched in MSC EVs, especially those from adipose tissue [229, 230]. These miRs inhibit the NF‐κB signaling pathway, thereby, reducing IL‐1β levels and alleviating inflammation in endothelial cells, ultimately promoting wound healing [229, 230]. MSC EVs also facilitate M2 macrophage polarization by delivering miR‐21‐5p, which suppresses kruppel‐like factor 6 (KLF6), thereby, enhancing anti‐inflammatory responses and promoting tissue repair [231]. Beyond their immunomodulatory properties, MSC EVs carry pro‐angiogenic VEGF and miR‐126, which promote neovascularization and alleviate oxidative stress in damaged tissues [232, 233]. Notably, miR‐26a‐5p, highly expressed in adipose‐derived MSC EVs, exhibits therapeutic potential in diabetic kidney models, both in vitro and in vivo, by targeting TLR4, suppressing NF‐κB signaling, and downregulating VEGFA expression [234]. Other studies have also shown that adipose‐derived EVs suppress the TLR4 pathway [235, 236], which is known to enhance SASP [218].

Several rodent studies have demonstrated that MSC EVs exert anti‐aging effects in the kidneys. For example, Yu and colleagues showed that naturally aged mice have reduced phosphorylation of pre‐lamin A/C in kidney tissue compared to young mice [237]. Their phosphoproteomic analysis further revealed that treatment with MSC EVs significantly increased phosphorylation of serine at position 390 and 392 in the amino acid chain of lamin A/C, which is associated with improved nuclear structure and delayed cellular senescence [237]. Moiseeva and colleagues reported that lamin A/C regulates cell cycle arrest and senescence via the p53‐p21 axis [238] and Yu and colleagues confirmed that MSC EVs modulate this pathway in aged kidneys. In addition, Dorronsoro and colleagues demonstrated that administration of MSC EVs in naturally aging mice suppressed cellular senescence and extended healthy lifespan [239]. It is worth noting that therapeutic mechanisms of EVs remain only partially understood. While EV biomolecules, such as proteins and RNA, have been shown to regulate aging and inflammation‐related pathways, the roles of lipids and carbohydrates, as well as the effects of the biomolecular corona formed or the surface of EVs, remain largely unknown [211, 240, 241, 242, 243].

Despite the promising effects of MSC EVs in kidney disease, therapeutic applications are limited by several constraints. For example, the biodistribution of MSC‐EVs is not kidney‐specific and kidney accumulation of EVs may only partially improve in pathological conditions, as the glomerular filtration barrier limits EV penetration in healthy kidneys [244, 245]. Additionally, repeated administration of EVs may elicit immune responses [246, 247]. However, findings suggest that EVs may be a less immunogenic alternative to cell therapy. In particular, when used as therapeutics in a mouse model of renal artery stenosis, MSCs strongly induced antibody production, whereas EVs exhibit lower immunogenicity within the same disease context [248].

Additional challenges in the field include the existence of multiple EV isolation, characterization, and storage protocols that vary in yield, purity, and risk of contaminant inclusion, such as lipoproteins, protein aggregates, and polysaccharides [249, 250, 251, 252]. Therefore, it is difficult to determine whether the observed therapeutic effects are due to the EVs themselves or to the contaminating components. Novel methods to remove contaminants have been proposed, including chemically‐induced lipoprotein breakdown [253]. The use of starvation or chemically‐defined media when culturing cells for EV production can eliminate chances of contamination with serum components, however, such conditions have limited physiological relevance [254]. It should also be noted that chemically‐defined media can give rise to protein aggregates that co‐isolate with EVs and contaminate samples [255]. To further complicate matters, some components previously considered contaminants, may from functional complexes with EVs [241, 256]. Additionally, the presence or absence of cryoprotectants during storage affects the preservation of EV membrane structures and functional effects [257, 258]. Such variability in methods greatly hinders the comparison of results and reproducibility across studies.

The clinical translation of EV‐based therapies also faces its own set of challenges, including donor heterogeneity [259]. Even with derived from the same donor, the intrinsic heterogeneity of EVs may impact batch‐to‐batch consistency, leading to variability in properties such as composition and purity, which affects safety and efficacy [204, 205]. Scalable manufacturing of EVs also remains a substantial challenge, although approaches, such as tangential flow filtration, are promising for processing large volumes of EV‐containing fluid [260, 261]. Additionally, ensuring storage stability and optimizing dosing regimens remain challenging.

To address these limitations and facilitate the successful clinical translation of MSC EV‐based therapies, future research should focus on several key areas. First, standardization of EV isolation, characterization, and storage protocols is essential to improve reproducibility and reduce batch‐to‐batch variability [262, 263, 264, 265]. Second, advances in EV engineering, including surface modification and optimized cargo loading, may enhance kidney‐specific targeting and therapeutic efficacy [266, 267, 268]. Third, scalable and good manufacturing practice (GMP)‐compliant manufacturing platforms are required for clinical‐grade EV production [262, 263, 269]. In addition, comprehensive evaluation of biodistribution, immunogenicity, and long‐term safety, particularly under repeated dosing regimens, will be critical [262, 269, 270]. Finally, improved mechanistic insights into how individual EV cargo components and the biomolecular corona contribute to therapeutic outcomes will support the rational design of next‐generation MSC EV‐based therapies [263, 266, 268].

## 6 Combination Senotherapeutics

### 6.1 EV‐Based Combination Strategies With Small‐Molecule Senotherapeutics

While modular use of senolytics, senomorphics, MSC EVs, and longevity‐enhancing proteins has yielded promising outcomes individually, emerging evidence suggests that combination therapies, targeting different, but complementary aspects of senescence, may offer synergistic benefits [273]. For example, combination therapies include pairing dasatinib with polyphenols, such as resveratrol or ellagic acid, demonstrating comparable or even superior efficacy to its conventional combination with quercetin. When screened in senescent human lung fibroblasts, dasatinib in combination with resveratrol or ellagic acid enhanced senotherapeutic effects relative to each compound alone [274]. Notably, ellagic acid was more potent than quercetin, allowing it to be used in lower doses, while resveratrol attenuated inflammatory cytokine release during the senolytic process [274]. Similarly, pulse administration of the senolytic alvespimycin, combined with ongoing treatment using senomorphic, GS‐444217, more effectively reduced tubular senescent cell burden and inflammatory markers in a diabetic acute kidney injury mouse model, over the senolytic alone [275]. These findings suggest that multitarget combination approaches using senolytics and senomorphics may be particularly effective and provide a conceptual framework for applying similar strategies to EV‐based therapies. However, although combination therapies can demonstrate synergistic efficacy, they also carry risks of additive or synergistic toxicities that may cause harm. Dasatinib's myelosuppression and pleural effusions combined with polyphenols' gastrointestinal/hepatic effects, or HSP90 inhibitors' hepatotoxicity paired with senomorphics' metabolic disruption, could amplify off‐target damage to healthy tissues, particularly in frail kidney disease patients [124]. Thus, safety studies with dosing considerations will be informative, as limited trials have been performed in the context of kidney disease. Preclinical data are mixed: alvespimycin with GS‐444217 showed no exacerbated toxicity beyond single agents in diabetic AKI [275], but other preclinical combinations of BCL‐2 family inhibitors with additional agents demonstrate increased cytotoxicity in non‐senescent cells, highlighting potential for compounded harm [276]. Mitigation strategies include (1) dose reduction (leveraging potency gains), (2) intermittent “hit‐and‐run” scheduling, and (3) EV‐mediated tissue‐specific delivery (67% uptake in injured vs. 36% healthy tubular cells), minimizing systemic exposure while concentrating therapeutics at senescent lesions [277].

### 6.2 EV‐Based Combination Strategies With Longevity Proteins

In parallel with small‐molecule approaches, EVs have also been explored as delivery vehicles for longevity‐associated proteins, offering both stabilization of therapeutic cargo and enhanced tissue‐specific targeting. Therapeutic EVs were shown to reduce inflammation, oxidative stress, and fibrosis while promoting tissue regeneration. However, existing data indicate that they only partially reverse established kidney damage and aging‐related changes, suggesting the potential need for combination strategies [271, 272]. Urine‐derived EVs carrying the endogenous longevity protein α‐klotho accelerated kidney recovery, reduced inflammatory and injury markers, and restored native α‐klotho levels in a mouse model of acute tubular injury [278]. Importantly, recombinant α‐klotho administered alone at a dose equivalent to that present within the EVs failed to produce comparable regenerative benefits [278], highlighting the additive and reparative effects conferred by EV‐mediated delivery. Likewise, *Kl*‐overexpressing MSC‐derived small EVs (α‐klotho‐sEVs) significantly accelerated kidney recovery, reduced injury and inflammation markers, and restored endogenous α‐klotho in rhabdomyolysis‐induced murine AKI, outperforming unmodified sEVs [279]. These findings emphasize the dual role of EVs as both active therapeutics and effective delivery vehicles [280, 281, 282]. Patel et al. further highlighted the importance of multitarget senotherapeutic and regenerative strategies in the treatment of diabetic kidney disease, supporting the rationale for combining EV‐based therapies with longevity‐enhancing proteins [273]. Taken together, these findings indicate that EV‐protein combination approaches may overcome limitations associated with protein instability, poor biodistribution, and limited tissue uptake.

### 6.3 Targeting Mechanisms and Biodistribution of MSC EVs in Kidney Injury

MSC EVs possess surface molecules that facilitate homing to sites of tissue damage and inflammation. Optical imaging studies using fluorescently‐labeled EVs in murine models of glycerol‐induced AKI have demonstrated that MSC EVs specifically accumulate in the kidneys of mice with AKI, while no signal was detected in the kidneys of healthy control mice [224]. Related studies have further characterized this targeting mechanism, showing that injured kidneys displayed fluorescence signals that were quantifiably higher than sham‐operated controls, and at the cellular level, damaged tubular epithelial cells demonstrated EV uptake efficiency of 67% compared to 36% in control cells [277]. This enhanced cellular uptake is mediated by injury‐induced upregulation of adhesion molecules (vascular cell adhesion molecule 1 and intercellular cell adhesion molecule‐1) in damaged tissue, which interact with corresponding integrins (very late antigen‐4 and lymphocyte function‐associated antigen 1) present on EV surfaces [277]. Quantitative analysis has confirmed that EV localization to damaged tissue exceeds that observed in healthy tissue, enabling this preferential targeting to concentrate therapeutic EVs at sites requiring regeneration while reducing distribution to healthy tissues [224, 277].

### 6.4 Engineering Strategies and Challenges for EV‐Based Combination Senotherapeutics

Beyond these intrinsic biological properties, EVs can also be further engineered to enhance therapeutic efficacy. For instance, EVs can be loaded with therapeutic small molecules or biologics either by engineering producer cells or by direct chemical or physical loading methods [283, 284]. Moreover, surface engineering of EVs can further enhance tissue specificity. For example, cluster of differentiation 38 (CD38)‐targeted MSC EVs have been used to deliver SIRT1 activators to senescent type 2 alveolar epithelial cells in a mouse model of pulmonary fibrosis, resulting in enhanced SIRT1 activity, improved mitochondrial function, and reduced fibrosis [285].

In the context of kidney disease, preclinical studies have begun to explore the combination of EVs with senolytic drugs or longevity proteins. For example, dasatinib‐ and quercetin‐loaded EVs can selectively target and eliminate senescent cells in murine models, enhancing tissue regeneration and reducing markers of senescence [286]. While direct evidence in chronic kidney disease models remains limited, these approaches provide a rationale for developing EV‐based combination therapies that can simultaneously modulate the senescence‐associated secretory phenotype (SASP), restore mitochondrial function, and enhance anti‐aging signaling pathways. Despite encouraging preclinical evidence, several challenges remain for EV‐based combination therapies, including cargo loading efficiency, heterogeneity of EV preparations, scalability of production, dose standardization, and long‐term safety [287, 288, 289, 290, 291, 292].

### 6.5 Perspective

Taken together, the combination of MSC EVs with small‐molecule senotherapeutics or longevity proteins presents a compelling therapeutic strategy for addressing cellular senescence in chronic kidney disease. In this best‐of‐both‐worlds approach, MSC EVs possess robust immunomodulatory and pro‐regenerative properties that can tackle the SASP secretome, while supplementary longevity proteins can counteract the under expression of native anti‐aging genes. The dual functionality of EVs as endogenous immunomodulatory agents and exogenous carriers for therapeutic cargo can be leveraged to provide a multifaceted strategy to mitigate disease progression in chronic kidney disease.

## 7 Conclusion

MSC EVs represent a promising and multifaceted therapeutic strategy for chronic kidney disease, targeting the complex interplay of inflammation, tissue injury, and cellular senescence. Inherent repair properties enable MSC EVs to alleviate tissue damage induced by the SASP, while concurrently exerting favorable immunomodulatory effects. Additionally, EVs have the ability to deliver therapeutic cargo, enabling combination therapies that target senescence. The incorporation of longevity proteins such as SIRT1 or α‐klotho into EVs, has potential to restore key anti‐aging signaling pathways, such as SIRT1 activation and Wnt/β‐catenin modulation, to counteract cellular senescence and promote tissue repair. Such combination therapies may enhance kidney regeneration and help preserve long‐term kidney function more effectively than monotherapies. Nevertheless, several challenges must be addressed to realize the clinical potential of EV‐based combination therapies. These include standardization of EV isolation, characterization, and storage protocols, development of scalable manufacturing processes, and thorough evaluation of immunogenicity, particularly in the context of repeated dosing. Additionally, further mechanistic studies are needed to elucidate the roles of EV cargo components, such as lipids, glycans, and the biomolecular corona, in mediating therapeutic effects and biodistribution. In conclusion, harnessing the dual functionality of MSC EVs as both endogenous modulators and exogenous delivery vehicles together with longevity proteins, offers a rational and innovative approach to address the multifactorial pathogenesis of chronic kidney disease. Continued research and technological advances will be essential to translate this potential into improved clinical outcomes for patients.

## Conflicts of Interest

The authors declare no conflicts of interest.
